# Indoor residual spraying with microencapsulated DEET repellent (N, N-diethyl-m-toluamide) for control of *Anopheles arabiensis* and *Culex quinquefasciatus*

**DOI:** 10.1186/1756-3305-7-446

**Published:** 2014-09-23

**Authors:** Jovin Kitau, Richard Oxborough, Johnson Matowo, Franklin Mosha, Stephen M Magesa, Mark Rowland

**Affiliations:** Department of Entomology and Parasitology, Kilimanjaro Christian Medical University College (KCMUCo), Moshi, Tanzania; Pan-African Malaria Vector Research Consortium, (PAMVERC), Moshi, Tanzania; Department of Disease Control, London School of Hygiene and Tropical Medicine (LSHTM), London, UK; National Institute for Medical Research, Dar es Salaam, Tanzania

**Keywords:** Indoor residual spraying, DEET, Repellent, *Anopheles arabiensis*, *Culex quinquefasciatus*

## Abstract

**Background:**

Evolution of insecticide resistance in *Anopheles gambiae* complex necessitates evaluation of alternative chemical classes to complement existing insecticides for long lasting insecticidal nets (LLIN) and indoor residual spraying (IRS). Microencapsulated (MC) DEET (N, N-diethyl-m-toluamide) is a formulation of the popular repellent, which gives long lasting activity when applied to nets. Its suitability for IRS use has not been evaluated before. This study assessed the efficacy of DEET MC, for IRS in experimental huts.

**Methods:**

DEET MC was tested alongside standard repellent and non-repellent residual insecticides: lambdacyhalothrin, permethrin, pirimiphos methyl and DDT. Residual formulations of these compounds were sprayed on plywood panels attached to walls of experimental huts to assess efficacy against pyrethroid resistant, wild free-flying *Anopheles arabiensis* and *Culex quinquefasciatus*. The panel treatments were rotated weekly between huts.

**Results:**

The overall mortalities of *An. arabiensis* induced by the various treatments (range: 76-86%) were significantly greater than mortality in the untreated control (8%, P < 0.001). Mortality of *An. arabiensis* in DEET sprayed huts (82%) was higher than in lambdacyhalothrin CS (76%, P = 0.043) but not significantly different to pirimiphos methyl CS (86%, P = 0.204) or DDT huts (81%, P = 0.703). Against *Cx. quinquefasciatus* DEET MC was less effective, inducing lower mortality (29%) than other treatments. *An arabiensis* blood feeding rates were higher in the unsprayed control (34%) than in sprayed huts (range between treatments: 19-22%, P < 0.002), and DEET provided equivalent or superior blood feeding inhibition (44%) to other insecticides. Against *Cx. quinquefasciatus* there was no significant reduction in blood-feeding for any treatment relative to the control. There was a significantly higher exiting of *An. arabiensis* from huts sprayed with DEET (98%), lambdacyhalothrin (98%) and permethrin (96%) relative to the control (80%, P < 0.01). Exiting rates of *Cx. quinquefasciatus* did not differ between treatment huts and the control.

**Conclusion:**

Microencapsulated DEET acts like an insecticide at ambient temperature and induces mosquito mortality when applied to walls made from wooden panels. This trial demonstrated the potential of microencapsulated DEET to control *An. arabiensis* and warrants further studies of residual activity on interior substrates.

## Background

Indoor residual spraying (IRS) has played an important role in reducing malaria transmission and morbidity and mortality in various endemic settings [1]. Insecticides like dichloro-diphenyl-trichloroethane (DDT) and benzene hexachloride (BHC) were successfully used in indoor residual spraying (IRS) during the 1950s and 1960s Malaria Eradication Programme. Significant reduction in malaria cases and vector densities were achieved in many parts of the world including, in the African continent, Southern Africa [2, 3]. Despite this contribution in the control and interruption of malaria, the use of IRS declined due to the decreased political, financial and technical commitment to the Malaria Eradication Programme [4] and to Stockholm treaty restrictions on the use of DDT [5]. The World Health Organization (WHO) has recently reaffirmed the importance IRS as a primary intervention for community protection in stable transmission settings and use of DDT for malaria vector control [6–8]. IRS is still effective and trials in Africa and Asia have led to a significant reduction in malaria prevalence and risk of being bitten by infective malaria mosquitoes. For example in Tanzania over 60% and 55% reduction in the prevalence of parasitaemia and anemia in children under five years of age were recorded respectively following two rounds of lambdacyhalothrin spray [9]. Sharp *et al.*, [10] showed reduction in sporozoite rates and numbers of susceptible *Anopheles gambiae s.s.*, *An. melas* and *An. funestus* in Equatorial Guinea after spraying of lambdacyhalothrin. In Malaysia, Rohani *et al*. [11] reported residual spraying of deltamethrin to be effective against indoor resting anophelines, and successfully reduced slide positivity rates and malaria cases by 90%-100%.

Insecticides other than pyrethroids and DDT have successfully been used for IRS against different mosquito species [12–14]. For example, two rounds of bendiocarb (carbamate) spraying in a community trial in Benin reduced biting rates of resistant *An. gambiae* by over 80% and parous rate by more than 60% [13]. Pirimiphos methyl (organophosphate) CS formulation sprayed on cement walls in experimental huts sustained the killing of over 80% of pyrethroid resistant *An. gambiae* for 9 months [14]. In Tanzania over 70% of *An. arabiensis* entering pirimiphos methyl sprayed experimental huts were killed 12 months after spraying [15]. Pirimiphos methyl has been used successfully in control programs in Malawi and Zambia [16].

Despite impressive results, a major problem threatening IRS is insecticide resistance, particularly pyrethroid-resistance, which is now widespread in *Anopheles* species across sub-Saharan Africa [17–24]. Additionally there are increasing reports of resistance to other classes of public health insecticide including carbamates and organophosphates [25–27]. As a result of insecticide resistance national control programmes are being forced to fall back on alternatives which are more expensive than pyrethroids for IRS [28, 29]. Only four classes of insecticide are recommended by The World Health Organization Pesticide Evaluation Scheme (WHOPES) for IRS and no new class has been developed for several decades due to the high cost of research and development and the comparatively small market for public health insecticides [30]. The Innovative Vector Control Consortium (IVCC) facilitates research and development on alternative insecticides and improved formulations of existing insecticides. The IVCC has been involved in successful development of long-lasting formulations of p-methyl and deltamethrin, both recently approved by WHO [31]. Because the emergence of new, safe classes of public health insecticide is inevitably slow [30, 32–35] there is a more urgent need to develop and evaluate new formulations of existing compounds which have potential for IRS in order to reduce overreliance on pyrethroids, carbamates and organophosphates.

DEET (N, N-diethyl-*m*-toluamide) is a highly effective, synthetic repellent used for topical skin application in varying formulations and concentrations and is considered the gold standard [36]. Complete protection for over 5 hours has been recorded against *Aedes aegypti* [37] and against *Anopheles gambiae* s.s, *An. albimanus* and *An. stephensi* with concentrations of 4 to 23% [38]. When used on a wide scale DEET topical application has been shown to reduce house resting *Anopheles gambiae* s.s. and *Anopheles arabiensis* in Tanzania and malaria incidence in Pakistan, [39, 40]. However, a trial of DEET in the Mekong region of SE Asia showed no reduction in malaria incidence [41]. Variation in effect is to be expected because regular use of topical repellent is highly dependent on individual discipline and cultural preferences [42].

DEET impregnated bed nets have been shown to have lethal effects on mosquitoes rather than repellent activity perhaps due to lower volatility at indoor ambient temperature [43–45]. With the development of a microencapsulated formulation to improve residual efficacy, DEET alone and in mixtures has been evaluated for mosquito control [45–47]. The toxic properties of DEET coupled with its repellency and irritancy makes it a potential indoor residual spray treatment [48]. This trial evaluated the effectiveness of DEET MC indoor residual spraying, relative to commonly used residual insecticides, in experimental huts for control of free-flying, wild *An. arabiensis* and *Cx. quinquefasciatus* mosquitoes.

## Methods

### Study site and experimental huts

The trial was conducted in experimental huts at the Pan-African Malaria Vector Research Consortium (PAMVERC) field station of Kilimanjaro Christian Medical University College (KCMUCo) in Lower Moshi, Kilimanjaro, Tanzania. The station is situated in Lower Moshi Rice Irrigation Zone (3°22′S, 37°19′E; altitude 800 m) where the mosquitoes *An. arabiensis* and *Culex quinquefasciatus* predominate [49]. The *An. arabiensis* are known to be resistant to pyrethroids but susceptible to carbamates and organophosphates [49, 50]. The huts were built to a design described by the World Health Organization [51] based on the original East African verandah-hut design [52, 53]. A significant modification was made to the design by installing wooden eave baffles on two sides (East and West) that allowed entry but prevented egress of mosquitoes that entered the huts. The other two eaves were left open (un-baffled) so mosquitoes could exit and subsequently be collected in screened verandahs. For the present study to enable rotation of treatments between huts all inner walls of experimental huts were covered with wooden panels on which the respective treatments were applied. Panels made of plywood were assembled and attached to walls using metal stoppers. The ceiling of each experimental hut room was covered with a plastic sheet to discourage mosquitoes from resting.

### Resistance tests

To confirm resistance status, samples of adult *An. arabiensis* and *Cx. quinquefasciatus* were collected from habitations in the vicinity of the trial site and subjected to resistance testing to 4 classes of insecticide in WHO kits as per WHO guidelines [54].

### Chemical formulations and dosages

The following insecticide formulations were used: DEET MC (Higashi-Nihombashi, Chuo-ku, Tokyo), lambdacyhalothrin CS (100 g/l) (Icon CS®, Syngenta, Basel, Switzerland), permethrin 50% EC (Sumitomo Chemical Co., Ltd., Tokyo, Japan), Pirimiphos methyl CS (300 g/l) (Actellic CS®, Syngenta, Basel, Switzerland) and DDT 750WP (Avima Ltd, Kenmare, South Africa).

As DEET has not been tested as IRS before, we selected an application rate known to be effective on other materials. A dosage of 8 g/m^2^ produced high levels of insect mortality on netting in laboratory and experimental huts tests and was not dissimilar to rates applied in topical applications to skin [43, 45, 55, 56]. This choice of application rate was made with the assumption that dermal exposure from wall application would be much lower than with normal topical application. Lambdacyhalothrin, permethrin, pirimiphos methyl and DDT were applied at dosages recommended by the World Health Organisation Pesticide Evaluation Scheme (WHOPES) [8, 57].

The following treatments were sprayed and evaluated in 6 experimental huts:

Unsprayed control

DEET MC 8 g/m^2^,

Lambdacyhalothrin CS 0.025 g/m^2^

Permethrin EC 0.5 g/m^2^

Pirimiphos methyl CS 1 g/m^2^

DDT WP 2 g/m^2^

### Treatment of panels

To avoid contamination, spraying of panels was done at a safe distance from the experimental hut area. The wooden wall panels, measuring 265 cm by 190 cm, were removed from experimental huts, temporarily erected on the inner walls of the isolated building, and sprayed using a Hudson X-pert sprayer (H.D. Hudson Manufacturing Company, Chicago, Ill. USA) with flat fan 8002E nozzle at an application rate of 40 ml/m^2^ [58]. Compression was maintained at 55 psi by re-pressurizing after each two swaths and flow rate was 840 ml/minute. Swath boundaries were marked with chalk and a guidance pole was used to ensure consistency and improve spray accuracy. To avoid any contamination after spraying the treatment panels were moved back to the huts to be assembled and the sprayer was thoroughly rinsed with water. Experimental huts were left over night to aerate before commencing the trial.

### Procedure

A suite of six huts were used for the trial. Adult volunteers slept in the huts from 19:30–6:30 hours. Each morning, mosquitoes were collected from the verandah and window traps of huts. White plastic carpets were laid on the floor to make dead mosquitoes more easily visible. Live mosquitoes in the room were not collected in order to allow for natural resting times on treated surfaces, and were only collected after exiting to verandah or window traps. Collected mosquitoes were recorded as blood-fed or unfed and as dead or alive. Live mosquitoes were kept in paper cups with 10% glucose solution for 24 hours before scoring delayed mortality. Collected mosquitoes were identified based on their morphological characteristics and grouped as either *Anopheles gambiae s.l*. or *Culex quinquefasciatus. Anopheles arabiensis* predominates in high altitude, low humidity areas and therefore the *An. gambiae s.l.* collected were assumed to be *An. arabiensis* based on earlier and recent PCR identifications [59]. Sleepers rotated between huts after each trial night to reduce any bias due to differences in individual attractiveness to mosquitoes. Treatments were rotated between huts every 7 days according to a Latin square design. On the rotation day control and other treatment panels were dismantled and taken outside before cleaning the huts. After cleaning, the huts were left for 2 days for airing before resuming the trial to allow time for any vapour from previous treatments to dissipate [51].

The primary outcomes were:

– Mortality (the proportion of mosquitoes killed out of total number collected).
– Blood feeding inhibition (the reduction in blood feeding in treatment huts relative to the control hut);

Secondary outcomes were:

– Induced exiting (the proportion of mosquitoes that were collected from exit traps and verandahs in treatment huts relative to control huts);
– Deterrence (percentage reduction in the number of mosquitoes found in a treated hut compared with the number in the control hut)

### Data processing and analysis

Data was entered into an Excel database and transferred to Stata® 10 (Stata Corp LP, College Station, TX, USA) for analysis. The outcomes of interest were proportion of mosquitoes dead (mortality), blood-fed and exiting on successive nights. Logistic regression for proportional data was used to estimate the outcomes, comparing results for treated and untreated huts, clustering by day, and adjusting for variation between individual sleepers and experimental huts. Estimated proportions were corrected for control mortality using Abbot’s correction. Insecticide induced exophily and blood feeding inhibition in treated huts were calculated using the respective untreated controls.

### Ethical approval

The study received approval from London School of Hygiene and Tropical Medicine and the National Ethics Committee of Tanzania (NIMR/HQ/R.8c/Vol. I/24). Details of the study were explained to all participating volunteers who gave their written consent. During the trial all volunteers were monitored each day for signs of fever or possible side-effects of the chemicals.

## Results

### Resistance tests

*An. arabiensis* were confirmed as resistant to pyrethroids but susceptible to carbamates and organophosphates. WHO tests with lambdacyhalothrin, malathion and DDT on local *Cx. quinquefasciatus* indicated resistance to pyrethroids and DDT but susceptibility to organophosphates (Table 1).

**Table 1:** **Resistance status of wild** ***Anopheles arabiensis*** **and** ***Culex quinquefasciatus*** **mosquitoes from lower Moshi (Mabogini) to pyrethroid, organophosphate and DDT WHO test papers**

Mosquito species	Insecticide treatment	No tested	% mortality (95% CI)
*An. arabiensis* ^*^	Permethrin (0.75%)	198	84 (78–89)
	Lambdacyhalothrin (0.05%)	100	74 (65–83)
	Malathion (5%)	202	100
	DDT (4%)	100	99 (97–100)
*Cx. quinquefasciatus*	Permethrin (0.75%)	100	68 (59–77)
	Lambdacyhalothrin (0.05%)	100	70 (60–80)
	Malathion (5%)	100	100
	DDT (4%)	100	48 (38–58)

^*^Matowo *et al.* [50].

### Experimental hut trial

#### Entry rates

A total of 2436 mosquitoes were collected over 36 nights. *Anopheles arabiensis* mosquitoes were over 3 times more abundant than *Culex quinquefasciatus* (77.5% and 22.5% respectively).

There were fewer *An. arabiensis* collected in control than in all treatment huts (Table 2). The total numbers of *Cx. quinquefasciatus* collected were lower in huts sprayed with lambdacyhalothrin, permethrin and p-methyl than that in unsprayed control hut. It was not appropriate to assess deterrence based on the finding of fewer *An. arabiensis* in the control, as hut proximity to breeding sites during the rotation was important. The number of *An. arabiensis* collected per week was 4–6 times more numerous during weeks 3–6 than during weeks 1–2.

**Table 2:** **Number of mosquitoes entering experimental huts (n = 36 nights)**

	Untreated control	DEET	Lambda-cyhalothrin	Permethrin	Pirimiphos methyl	DDT
***Anopheles arabiensis***						
Total females caught	165	362	348	415	306	292
Females caught/night	5^a^	10^a^	10^a^	11^a^	8^a^	8^a^
***Culex quinquefasciatus***						
Total females caught	109	117	62	84	76	100
Females caught/night	3^a^	3^a^	2^a^	2^a^	2^a^	3^a^

Numbers in the same row sharing a letter superscript do not differ significantly (*P* > 0.05).

#### Mortality rates

**Figure 1:**
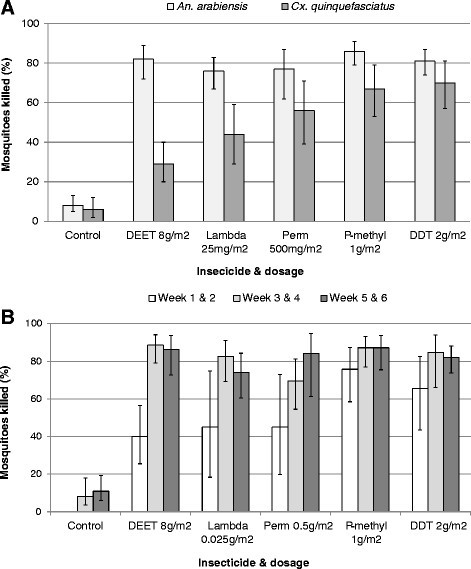
**Percentage mortality of both Anopheles arabiensis and Culex quinquefasciatus by treatment (A) and Anopheles arabiensis alone over the trial period (B).**

The overall mortality of *An. arabiensis* collected in huts was high for all treatments and was 10 times greater than the mortality observed in the unsprayed control huts (Figure 1A, Table 3). Overall mortality in DEET sprayed huts (82%) was significantly higher than lambdacyhalothrin (76%, P = 0.043) and not statistically different to pirimiphos methyl (86%, P = 0.204). Mortality rates of *An. arabiensis* were lower during weeks 1–2 than during weeks 3–6 but this trend was consistent across all treatments and did not depend on active ingredient (Figure 1B).

**Table 3:** **Mortality of mosquitoes to IRS chemical treatments in experimental huts (n = 36 nights)**

	Untreated control	DEET	Lambda-cyhalothrin	Permethrin	Pirimiphos methyl	DDT
***Anopheles arabiensis***						
Total females dead	13	298	265	319	263	237
Mortality corrected for control (%)	-	74^bd^	68^c^	69b^c^	78^d^	73^bcd^
Unfed mortality	9^a^ (5–16)	85^bc^ (75–92)	80^b^ (71–86)	82^b^ (69–90)	88^c^ (82–93)	85^bc^ (77–91)
Blood fed mortality	5^a^ (2–14)	71^bc^ (55–83)	63^b^ (48–76)	59^b^ (37–78)	81^c^ (70–89)	66^b^ (57–74)
***Culex quinquefasciatus***						
Total females dead	6	34	27	47	51	70
Mortality corrected for control (%)	-	24^b^	39^bd^	51^cd^	63^c^	65^c^
Unfed mortality (95% CI)	4^a^ (1–17)	33^b^ (23–45)	49^bc^ (30–68)	63^cd^ (47–76)	71^d^ (53–85)	77^d^ (65–86)
Blood fed mortality (95% CI)	5^a^ (1–22)	20^b^ (9–41)	32^bc^ (15–55)	29^bc^ (12–57)	55^c^ (26–81)	45^c^ (22–71)

Numbers in the same row sharing a letter superscript do not differ significantly (*P* > 0.05).

Mortality rates of *Cx. quinquefasciatus* in all sprayed huts were much lower than those recorded for *An. arabiensis* (Figure 1A, Table 3). However, mortality rates associated with all sprayed huts were significantly greater than the control (P < 0.001). In contrast to *An. arabiensis* DEET sprayed huts produced the lowest mortality rates (29%) of any treatment but this was still significantly higher than the control (5%, P < 0.001). Consistent with *An arabiensis*, mortality rates were highest in huts with DDT (70%) and p-methyl (67%, Table 3).

For both species the mortality rates were higher among unfed mosquitoes than blood fed mosquitoes.

#### Blood-feeding rates

With the exception of p-methyl CS there was significant blood feeding inhibition in *An. arabiensis* across all treatments (35-44%) relative to the unsprayed control (P < 0.002, Table 4). Blood feeding inhibition was similar for DEET, lambdacyhalothrin, permethrin and DDT at 35-44%. *Culex quinquefasciatus* differed from *An. arabiensis* in that there was no significant reduction in mosquito blood-feeding for any treatment relative to the control (P > 0.05) with the exception of permethrin (P = 0.037).

**Table 4:** **Blood feeding rates of mosquitoes to IRS chemical treatments in experimental huts (n = 36 nights)**

	Untreated control	DEET	Lambda-cyhalothrin	Permethrin	Pirimiphos methyl	DDT
***Anopheles arabiensis***						
Total females blood fed	56	69	76	91	100	59
Blood fed% (95% CI)	34^a^ (22–49)	19^b^ (14–26)	22^b^ (16–29)	22^b^ (16–29)	33^a^ (25–41)	20^b^ (14–28)
Blood feeding inhibition (%)	-	44	35	35	2	41
***Culex quinquefasciatus***						
Total females blood fed	37	35	19	17	20	22
Blood fed % (95% CI)	34^a^ (23–46)	30^ab^ (20–42)	31^ab^ (20–43)	20^b^ (11–34)	26^ab^ (15–42)	22^ab^ (13–35)
Blood feeding inhibition (%)	-	12	9	41	24	35

Numbers in the same row sharing a letter superscript do not differ significantly (*P* > 0.05).

#### Exiting rates

In the control hut, over 80% of all *An. arabiensis* mosquitoes were collected from the screened veranda and exit traps indicating a high level of exophily (Table 5). However, there was a significantly greater level of exiting in the huts sprayed with DEET 98% (P < 0.001), lambdacyhalothrin 98% (P < 0.001) and permethrin 96% (P = 0.001) than the untreated control. Huts with p-methyl had the lowest mosquito exit rates (68%), presumably due to the large number of mosquitoes collected dead inside the room. Surprisingly, there was no significant difference between DDT (90%) and the control huts (88%, P = 0.681, Table 5).

**Table 5:** **Mosquitoes exiting from experimental huts (n = 36 nights)**

	Untreated control	DEET	Lambda-cyhalothrin	Permethrin	Pirimiphos methyl	DDT
***Anopheles arabiensis***						
Total females exiting	146	356	341	398	209	262
Exiting rate % (95% CI)	88^a^ (81–93)	98^b^ (94–100)	98^b^ (96–99)	96^b^ (94–97)	68^c^ (60–76)	90^a^ (85–93)
***Culex quinquefasciatus***						
Total females exiting	92	108	56	74	52	89
Exiting rate % (95% CI)	84^a^ (73–92)	92^a^ (82–97)	90^a^ (76–97)	88^a^ (77–94)	68^b^ (51–82)	89^a^ (79–95)

Numbers in the same row sharing a letter superscript do not differ significantly (*P* > 0.05).

Trends in exiting rates of *Culex quinquefasciatus* were similar to *An. arabiensis* except that the insecticide induced exiting tended to not be significantly different from the control (Table 5). Exiting ranged between 68-92%, with no difference between the control (84%) and DEET (92%, P = 0.068), lambdacyhalothrin (90%, P = 0.280), permethrin (88%, P = 0.465) and DDT (89%, P = 0.332). Huts sprayed with p-methyl (68%) had a significantly lower exit rate compared with the controls (84%, P = 0.011 Table 5).

## Discussion

The repellent properties of topically applied DEET are well known [60, 61] but this is the first report on the toxic effect of DEET when sprayed as a residual compound on intradomiciliary substrates. The experiment reported here shows that mortality of DEET against *Anopheles arabiensis* was equivalent to that shown by formulations of the organophosphate pirimiphos methyl and organochloride DDT, and was significantly higher than a residual pyrethroid lambdacyhalothrin. *An. arabiensis* from this study site expresses low level resistance to pyrethroids mediated through elevated levels of cytochrome P450s detoxification enzymes [62]. The high mortality for huts sprayed with permethrin and lamdacyhalothrin in the present study indicates that resistance is not yet of operational importance [63, 64].

Previous studies using DEET treated netting demonstrated toxic effects against Anopheline mosquitoes [43, 44]. N’Guessan *et al*. [43] showed that the majority of *An. gambiae* entering experimental huts and *Culex quinquefasciatus* in tunnel assays were killed by 8 g/m^2^ DEET treated nets. In the present IRS trial the insecticides used for comparison with DEET are recommended by WHOPES for indoor residual spraying for malaria control. Pirimiphos methyl, DDT and lambdacyhalothrin have been used successfully in the reduction of malaria transmission and mosquito vector density in several malaria endemic settings [65–68]. Lambdacyhalothrin has been used as part of the national control program in Tanzania from 2007 to present and intensively elsewhere in PMI-funded IRS campaigns throughout Africa. Therefore, DEET outperforming lambdacyhalothrin and its similar performance with WHOPES recommended insecticides highlights its potential for use as an IRS chemical.

Before DEET can be considered for IRS further studies are needed to determine the longevity of its residual activity on common household substrates. In this study the mortality effect in all treatments was low in the first two weeks, which coincided with lower mosquito numbers in the field. This low mortality in the first two weeks is perhaps due to repellence and/or irritancy of chemicals on freshly sprayed panels; mosquitoes entering during this period did not contact treated surfaces for long enough to pick up a lethal dose. Nevertheless there was no loss of activity on wood over the six week trial. Microencapsulation of DEET most definitely prolongs the residual properties of DEET on bed nets as compared to water-miscible lotion [45]. Mud and concrete are known to be more challenging substrates for IRS owing to sorption and alkaline pH and will have to be tested before a full recommendation can be made.

For *Culex quinquefasciatus*, mortality rates were lower in DEET sprayed huts than for other treatments. Although mortality in DEET sprayed huts was statistically higher than in control huts it remained surprisingly lower than other insecticide treatments unlike in previous studies elsewhere [43]. *Cx. quinquefasciatus* from the study area was resistant to pyrethroids in WHO susceptibility tests. This explains the low mortality rates observed for this species in huts treated with lambdacyhalothrin and permethrin. It is unlikely that the low mortality of *Cx. quinquefasciatus* recorded with DEET is due to a cross-resistance with pyrethroids. Toxicity of DEET is poorly understood. There is evidence that DEET can inhibit acetylcholinesterase and prevent hydrolysis of acetylcholine [69]. Organophospates and carbamates are notable acetylcholinesterase inhibitors but cross resistance to DEET through insensitive acetylcholinesterase *Ace-1*^*R*^ mechanism has not been documented [47, 70]. Rather, the low mortality in the present study could be due to repellence, meaning that *Cx. quinquefasciatus* did not contact treated surfaces for long enough to pick up a lethal dose or that the initial application dosage of 8 g/m^2^ was too low for this species.

Similar to lambdacyhalothrin, permethrin and DDT, DEET was observed to elicit reduced levels of blood-feeding against *An. arabiensis*. Protection against blood feeding in sprayed huts is a result of repellency and/or irritancy after mosquitoes contact sprayed surfaces. However, compared to the untreated control fewer *An. arabiensis* exited the pirimiphos methyl CS sprayed hut. This reduced exiting and the lack of protection against blood feeding in *An. arabiensis* coupled with high mortality rates indicates that pirimiphos methyl is primarily toxic, presumably with rapid toxicity preventing exiting.

In the sprayed huts presumably a proportion of *An. arabiensis* first landed on the walls and was repelled out of the huts before dying in the verandah unfed, having picked a lethal dose. This would explain the blood-feeding inhibition and greater degree of mortality in unfed than blood fed mosquitoes in all the treatments. Permethrin was the only treatment which reduced blood-feeding in *Cx. quinquefasciatus* compared to control and exit rates were no higher for sprayed huts than the control. The apparent lack of repellency resulting in no increase in exiting appears to explain the lack of blood-feeding inhibition for *Cx. quinquefasciatus*.

Chemical compounds for mosquito control are known to have multiple attributes and cannot be simply defined as either repellents or mortality inducing [71–75]. The actions of chemicals (toxicity, repellency or irritancy) differ with doses, chemical class and even between chemicals of same class. DDT is a prime example of an excito-repellent chemical with spatial repellency as its first and toxicity the third order mode of action [74]. Organophosphates on the other hand are primarily toxic with limited behavioural modifying responses at high doses [75]. Our results concur with findings from Tananchai [48] and Grieco [73] that DEET has multiple actions acting as an irritant/repellent and a toxicant. Results from this study did not record any differences between DDT, pyrethroids, and DEET in terms of chemically induced mosquito exit rates in *An. arabiensis*. This study did not assess spatial deterrence of mosquito entry in sprayed relative to the control huts due to the unexpected finding that far lower numbers of *An. arabiensis* were caught in control huts. This could be due to the positional bias of experimental huts, resulting in some huts being closer to breeding or resting sites. Treatments were rotated every 7 days to adjust for any bias, but in rice growing areas short peaks in numbers can occur, which may have skewed the numbers collected per treatment. Contamination of the control hut with residual vapors is unlikely because huts were thoroughly cleaned and left to aerate for 2 consecutive days during rotation. Another possible scenario is that mosquitoes escaped through small door or eave spaces. If this is true mortality in the treatments may have been overestimated (i.e. more mosquitoes collected in treated huts because they were killed before escaping). However, the effect of DEET should be judged comparatively and as it has been observed it matched the performance of WHOPES recommended insecticides.

Elsewhere in a village scale trial South-eastern Tanzania 100% coverage of DEET topical lotion reduced indoor resting mosquitoes by more than 60% compared to non-users [39]. If DEET were used as a high coverage IRS treatment it would be interesting to determine the contribution of spatial repellence against wild mosquito population and determine the relative importance of repellency, irritancy and toxicity. Two explanations have been given for the mode of action of DEET causing repellency. First, DEET is detected by mosquitoes through an activation of certain olfactory receptor neurons, to which mosquitoes respond by evasion [76]. Second, DEET modulates the function of olfactory receptor neurons in detecting and responding to host attractive odours [77]. As DEET has a different target site through olfactory receptor neurons for repellency, even if resistance to toxicity did develop, repellency may still provide some level of protection [76, 77], although, DEET insensitivity has been documented in *Aedes aegypti* mosquitoes [78].

Safety is a critical issue, and in recent years concerns have been raised over perceived risk of seizure following DEET exposure [79]. A 2010 EU Directive based on animal models recommends restricting concentration of DEET repellents to 15% or less [80]. However, DEET has been used as a topical repellent since registration in 1957 and despite >200 million applications annually, reports of adverse effects are low, with risk of seizure reported to be 1 per 100 million users [81]. There is no evidence to support increased risk in young children or pregnant women [82]. DEET IRS is likely to have a very low human safety risk, as application to walls and ceiling would result in much lower dosage of dermal or oral exposure compared to topical application.

The global plan for insecticide resistance management recommends rotation and/or mixtures of available insecticides where insecticide residual spraying remains effective [24]. However, from the WHOPES recommended chemicals for indoor spraying there are few insecticides to choose. DDT use should be eliminated as stipulated in the Stockholm Convention, and pyrethroid IRS should not be used where pyrethroid LLIN coverage is high. This leaves only carbamates and organophosphates, which severely limits insecticide resistance strategies. The development of new active ingredients is slow. Despite added momentum from the IVCC only p-methyl CS and deltamethrin SC-PE have been recommended in the past decade [14, 15]. This proof of concept study has demonstrated that DEET kills high proportions of *An. arabiensis* in experimental huts for 6 weeks. More work is needed to determine the efficacy and residual activity of DEET against *An. gambiae s.s.* and *An. funestus* and comparing efficacy on different substrates types used in housing structures.

## Conclusions

Previous studies had shown DEET to be a repellent and toxicant to mosquitoes when used in topical applications and in impregnating fabrics respectively. The present study is the first to demonstrate that DEET can be highly effective as a residual insecticide, in terms of killing and blood-feeding inhibition, against pyrethroid resistant *Anopheles arabiensis* when sprayed on wooden substrates. DEET IRS produced levels of *An. arabiensis* mortality equivalent to that of pirimiphos methyl and DDT, and significantly higher than a residual pyrethroid lambdacyhalothrin. Additionally, DEET reduced blood-feeding against *An. arabiensis* to a similar degree as lambdacyhalothrin, permethrin and DDT. The comparable performance of DEET with WHOPES recommended insecticides highlights its potential for use as an IRS chemical. Nevertheless, before DEET can be considered for IRS further studies are needed to determine its efficacy against *An. gambiae s.s.* and *An. funestus* and longevity on different substrate types used in housing structures. The low mortality rates and lack of blood-feeding inhibition of DEET at 8 g/m^2^ against *Cx. quinquefasciatus* may be dosage related and warrants further investigation. While there have been increased concerns in recent years over the safety of DEET, its application to walls and ceiling would result in much lower dosage of dermal or oral exposure compared to topical application.
